# Power Analysis of C-TDT for Small Sample Size Genome-Wide Association Studies by the Joint Use of Case-Parent Trios and Pairs

**DOI:** 10.1155/2013/235825

**Published:** 2013-05-02

**Authors:** Farid Rajabli, Gul Inan, Ozlem Ilk

**Affiliations:** ^1^Department of Electrical and Electronic Engineering, Faculty of Engineering, Turgut Ozal University, 06010 Ankara, Turkey; ^2^Department of Statistics, Faculty of Arts and Sciences, Middle East Technical University, 06800 Ankara, Turkey

## Abstract

In family-based genetic association studies, it is possible to encounter missing genotype information for one of the parents. This leads to a study consisting of both case-parent trios and case-parent pairs. One of the approaches to this problem is permutation-based combined transmission disequilibrium test statistic. However, it is still unknown how powerful this test statistic is with small sample sizes. In this paper, a simulation study is carried out to estimate the power and false positive rate of this test across different sample sizes for a family-based genome-wide association study. It is observed that a statistical power of over 80% and a reasonable false positive rate estimate can be achieved even with a combination of 50 trios and 30 pairs when 2% of the SNPs are assumed to be associated. Moreover, even smaller samples provide high power when smaller percentages of SNPs are associated with the disease.

## 1. Introduction

Over a decade, there has been a considerable interest in identifying genes involved in diseases such as cardiovascular diseases, epilepsy, sleep disorders, and any kinds of cancer. In this sense, genetic association studies are extensively carried out to detect an association between a susceptible gene and the disease of interest. Furthermore, recent advances in biotechnology lead to genome-wide association studies (GWAS) and, in turn, enable genotyping hundreds of thousands of single nucleotide polymorphisms (SNPs) for a human genome [1]. Thus, GWAS make it possible to find out more genes associated with the disease under the concern than the expected.

Currently, genetic associations are tested through either population-based or family-based association studies, which are the two main groups of the association study designs. The most common and basic type of the population-based association studies is the classical case-control studies of unrelated affected and unaffected individuals [1]. However, one of the disadvantages of this kind of studies is that they may yield misleading inference in the presence of population stratification. On the other hand, the family-based association studies using any kind of pedigree receive attention since they are robust to population stratification if family information is properly incorporated into the analysis [2, 3]. 

Among family-based association study designs, transmission/disequilibrium test (TDT) [4] is the most widely used one [5]. However, this test requires a large number of mother-father-offspring trios to attain a reasonable statistical power. Recently, an alternative procedure, which is based on sequential probability ratio tests (SPRTs), is proposed to overcome this problem of limited sample size [6]. Through simulation studies, it was shown that SPRT resulted in smaller rates of false positives and negatives and higher accuracy and sensitivity values compared to TDT. 

In studies including only one affected offspring (case) with his/her two parents, who are all genotyped at a bi-allelic marker locus, TDT requires the genotype of both parents to be known. Similarly, SPRT was also proposed for the complete trios situation. However, in practice, contrary to what these tests require, it is possible to encounter circumstances when no genotype information is available for one or both of the parents. For instance, parental genotype information can be missing, especially in adult-onset diseases due to the fact that one of the parents may already be dead [5]. In such cases, besides case-parent trios, it is possible to see case-parent pairs. Case-mother pairs are the families in which genotypes of the mother and the affected child are available; case-father pairs are the families in which genotypes of the father and the affected child are available. 

In the case of a study which includes a combination of both family groups, ignoring families of pairs from the statistical analysis would lead to loss of information, which in turn would lead to loss of power [5, 7]. For that reason, it is very important to evaluate the information from both trios and pairs to maximize the utility [7].

In this sense, Allen et al. [8] proposed a combined transmission disequilibrium test (C-TDT) statistic to test for association when genotype information of both trios and pairs is available. Actually this test statistic is an extension of 1-TDT [9] so that the contribution of trios and that of pairs to the C-TDT statistic are the same. However, the proposed C-TDT statistic does not belong to a parametric statistical distribution under the null hypothesis of no association. Following to this, Hu and Zhou [7] suggested a permutation procedure for C-TDT to assess the significance.

The simulation studies in Hu and Zhou [7] showed that the use of C-TDT increases the statistical power, compared to TDT, in the presence of both trios and pairs. However, their work involves only a few SNPs. Since C-TDT is based on a permutation procedure, rather than an asymptotic statistical distribution, it is expected to be efficient even in small samples [10]. However, their study does not answer the question of how many trios and pairs are enough to attain a reasonable statistical power. Indeed, a common and important question in a study design is the determination of either the power of a statistical test for a pre-determined sample size or the required sample size to attain a certain power for that test.

In this paper, we aim to compute the power and false positive rate of the C-TDT statistic across different sample sizes in a family-based genome-wide study through simulation, so that this study would be a guide for a practitioner of C-TDT statistic to get an idea about either the power of the test for a predetermined sample size or to determine the required sample size to attain a certain power. Therefore, we aim to contribute in two aspects. First, we shed a light on the sample size needed for such a study. Second, we extend the computational work of Hu and Zhou [7] for thousands of SNPs. By this way, now it is possible to study family-based associations even in the case of small sample sizes with one missing parent. 

## 2. Method

In this section, we first review C-TDT and the permutation procedure. The readers who are familiar with these topics can skip to our simulation scenario in Section 2.3. 

### 2.1. C-TDT

**Figure 1:**
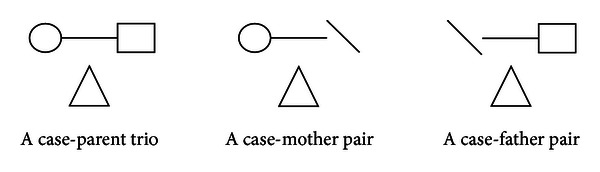
Representation of family types.

Although detailed statistical background information on C-TDT is given in Hu and Zhou [7], we believe that revisiting the formulation of C-TDT would be useful for the reader. In this sense, to display the C-TDT test statistic in a scenario, first of all, consider a family-based study, which consists of both trios and pairs with only one affected offspring as seen in Figure 1. In this figure, mother, father, and affected offspring genotypes are denoted by a circular, square, and triangle, respectively. Moreover, the “∖” sign indicates that the genotype is not available. 

Furthermore, assume that the marker locus is a bi-allelic with A and B alleles so that the genotype of each mother, father, and the affected offspring can be represented by AA, AB, or BB at any SNP. Then, the formulation of C-TDT can be given as

(1) C-TDT=[T−NT+ω(NM<C−NM>C)+(1−ω)(NF<C−NF>C)]2T+NT+ω2NM≠C+(1−ω)2NF≠C.

For case-parent trios, among the heterozygous parents, *T* is equal to the total number of mother and father, who transmits “A” allele but does not transmit “B” allele to the affected offspring and *NT* is the total number of mother and father, who does not transmit “A” allele, but transmits “B” allele to the affected offspring.

For families with only one parent available,  *ω* = *n*_*p*_/(*n*_*m*_ + *n*_*p*_), where *n*_*m*_ = the  number  of  case-mother  pairs and *n*_*p*_ = the number of case-father pairs. Here,  *N*_*M*<*C*_ = ∑*I*_*M*<*C*_ = the number of case-mother pairs in which the mother carries fewer copies of “A” allele than the case. On the other hand, *N*_*M*>*C*_ = ∑*I*_*M*>*C*_ = the number of case-mother pairs in which the mother carries more copies of “A” allele than the case. Here, *I* is an indicator variable taking the value of 1 when the statement holds and 0 otherwise and the summation is taken over all case-mother pairs. The numbers *N*_*F*<*C*_ = ∑*I*_*F*<*C*_ and *N*_*F*>*C*_ = ∑*I*_*F*>*C*_ can be defined in a similar fashion, where the summation is taken over case-father pairs. Finally, *N*_*M*≠*C*_ = *N*_*M*<*C*_ + *N*_*M*>*C*_ and *N*_*F*≠*C*_ = *N*_*F*<*C*_ + *N*_*F*>*C*_.

### 2.2. Permutation Procedure

As stated earlier, the distribution of C-TDT under the null hypothesis of no association cannot be clearly defined. One of the approaches to this problem would be to compute the significance of C-TDT through a permutation procedure. Actually, the permutation procedure can be used for any alleles test to obtain a *P* value, since this procedure does not require any assumptions such as variance assumptions or Hardy-Weinberg equilibrium (HWE) [11].

As Laird and Lange [11] stated the applicability of permutation procedures depends on the design of the study. Furthermore, the number of SNPs in the study increases the computational burden. Although the permutation procedure is an extensively used issue in case-control studies and software such as PERMORY [12], PRESTO [13], and PLINK [14] is developed, the use of permutation procedure in family-based designs is not as easy as in case-control studies. Therefore, more attention is required to apply the permutation procedure in family-based studies.

In this sense, Hu and Zhou [7] made use of the randomization procedure of Zhao et al. [15] for trio genotype data and they also proposed their own procedure for pair genotype data. They provided the necessary statistical theory behind the permutation of C-TDT. Their study was based on haplotype genotype data and they applied C-TDT only on a few SNPs. 

We can review and extend the permutation procedure of C-TDT for a GWA study for thousands of SNPs as follows.

(1) For a SNP, compute the C-TDT statistic through the original sample and save the result as the observed C-TDT.
(2) Choose the number of permutation samples large enough such as *R* = 1000.
(3) For each permutation sample, now there are four different steps to follow.
  (a) For the part of case-parent trios, one of all possible genotypes at that SNP is randomly assigned with equal probabilities as the genotype of the affected offspring.
  (b) For the part of case-parent pairs, permutation consists of the following series.
    (i) Let {(*C*_*i*_, *M*_*i*_), 1 ≤ *i* ≤ *n*_*m*_} be the genotypes of *n*_*m*_ case-mother pairs and similarly {(*C*_*i*_, *F*_*i*_), *n*_*m*_ + 1 ≤ *i* ≤ *n*_*m*_ + *n*_*p*_} be the genotypes of *n*_*p*_ case-father pairs, where *C*_*i*_/*F*_*i*_/*M*_*i*_ is the genotype of the case/father/mother in the *i*th case-parent pair, if any, respectively.
    (ii) Let {*j*_1_,…,*j*_*n*_*m*__, *j*_*n*_*m*+1__,…, *j*_*n*_*m*_+*n*_*p*__} be a permutation of {1,…, *n*_*m*_, *n*_*m*_ + 1,…, *n*_*m*_ + *n*_*p*_}, and then create the permuted *n*_*m*_ case-mother pair genotypes by
      (2) (Ci∗,Mi∗)={(Cji,Mji),if  ji≤nmfor  1≤i≤nm(Fji,Cji),if  ji>nm
     and create the permuted *n*_*p*_ case-father pair genotypes by
      (3) (Ci∗,Fi∗)={(Cji,Fji),if  ji>nmfor  nm+1≤i≤nm+np(Mji,Cji),if  ji≤nm.
  (c) Then, combine the case-parent trio genotypes created in (a) and the *n*_*m*_ case-mother pairs {(*C*_i_*, *M*_i_*), 1 ≤ *i* ≤ *n*_*m*_} and *n*_*p*_ case-father pairs {(*C*_i_*, *F*_i_*), *n*_*m*_ + 1 ≤ *i* ≤ *n*_*m*_ + *n*_*p*_} created in (b) to obtain “a complete permuted sample.”
  (d) Compute the C-TDT statistic for the permuted sample.
(4) Repeat step (3) for *R* = 1000 times and obtain the empirical distribution of the original test statistic such that {C-TDT_1_*, C-TDT_2_*,…, C-TDT_1000_*}.
(5) Compute, the *P* value of the observed C-TDT statistic, which is equal to the proportion of total number of C-TDT* values that are greater than the observed value of C-TDT:
  (4) P value=#(C-TDT∗>Original  C-TDT)1000.
(6) Repeat steps 1–5 for all SNPs to achieve a genome-wide study.

### 2.3. Simulation Scenario

A very common question that comes to mind prior to planning a study is either the power of a statistical test for a predetermined sample size or the required sample size to attain a certain power for that test. As a consequence of this, for family-based association studies, software such as PBAT [16] and TDT Power [17] calculator are developed. Accordingly, we believe that this study will be a guide for a practitioner of C-TDT statistic to get an idea about the relation between the power of C-TDT with a given sample size so that he/she can make a budget.

In this study, a Monte Carlo simulation study is carried out through C programming language to calculate the power of the C-TDT across different sample sizes in a family-based genome-wide study. In this context, it is decided to take 7 different sample sizes, such as 10 trios and 4 pairs, 30 trios and 18 pairs, 50 trios and 30 pairs, 70 trios and 40 pairs, 90 trios and 55 pairs, 110 trios and 65 pairs, and 130 trios and 75 pairs. Moreover, under any sample size, it is assumed that only 262,264 SNPs are observed for each experimental unit so that it can be representative of a GWAS. This is the number of SNPs that Affymetrix GeneChip Human Mapping 250K Nsp array covers. Following Hu and Zhou [7], the number of permutation replications is taken to be *R* = 1000. Finally, the simulation is repeated 100 times for each sample size. 

Under any sample size, at any simulation repetition, the generation of genotype information of trios and pairs is held separately as defined in Section 2.2.

First of all, for trios, the genotype information of the required number of mothers and fathers is generated randomly across 262,264 SNPs. In other words, for each SNP, parents are randomly assigned to one of the genotypes, AA, AB, or BB, with 1/3 probabilities.  Table 1 is expected to be utilized to determine the possible genotypes for an offspring of these parents. For instance, if the genotypes of both mother and father are AA, then the genotype of offspring can only be AA. It is assumed that common variants exist, and hence data are generated to provide minor allele frequencies greater than 5%.

To perform the power analysis, the genotypes of an offspring for 98% or 99% of 262,264 SNPs are imposed to be generated under *H*_0_. There is no association. For such SNPs, C-TDT statistics are expected to take small values. We make use of genotypic risk ratio (GRR) concept, which will be discussed in the coming paragraph, while deciding on the generation of SNPs. In this sense, when compared with other genotypes, relatively high probabilities are assigned to the offspring genotypes, whose family contributes to (1) as *T* = 0  and  *NT* = 0 or *T* = 1  and  *NT* = 1, as seen in Table 2. For example, as seen in Table 1, observing both mother and father genotypes as AB would normally lead us to assign 0.5 as the probability of obtaining an offspring with AB genotype and 0.25 as the probability of obtaining an offspring with either AA or BB genotypes. However, to generate in favor of *H*_0_, we assign 0.7 for the probability of obtaining an offspring with AB genotype and 0.15 for the probability of obtaining an offspring with AA or BB genotypes.

On the other hand, the genotypes of an offspring for 1% or 2% of 262,264 SNPs are forced to be generated under *H*_1_. There is significant association. As a consequence, similarly, relatively high probabilities are assigned to the offspring genotypes, whose family contributes to the statistic in (1) as *T* = 1 and *NT* = 0 or *T* = 2 and *NT* = 0, as seen in Table 2. For instance, observing the genotype of mother as AA and that of father as AB leads us to assign 0.7 for the probability of obtaining an offspring with AA genotype and 0.30 for the probability of obtaining an offspring with AB genotype. Obviously, the cases for which *T* = 0  and  *NT* = 1 or *T* = 0  and  *NT* = 2 would also result in an increase in C-TDT. However, we preferred to generate in only one direction to avoid the effect of one trio canceling out the effect of the other. 

The distribution for the genotypes of children is assumed skewed based on GRR. It was suggested that GRR can be approximated by *T*/*NT* [18, 19]. Under the null hypothesis, *T* and *NT* are equally likely, and hence the GRR is close to 1. Therefore, to generate data under the null hypothesis, we assign higher probabilities (e.g., 0.7) to the situations that lead to *T* = *NT*, and smaller probabilities (e.g., 0.15) to the cases with *T* ≠ *NT*. As the GRR shifts from 1, the association between the disease and the marker is expected to increase, and hence, we are more likely to reject  *H*_0_. Kharrat et al. [19] reported that in complex diseases, most associated genes have low or medium GRR values (between 1.5 and 3.5). Following them, we assign higher probabilities to the cases with *T* > *NT* to obtain GRR > 1.5 under  *H*_1_. For instance, assigning 0.7 probabilities to observe an AA offspring from an AA mother and AB father is expected to lead to a GRR ≈ 0.7/0.3 ≈ 2.3. 

Accordingly, for pairs, the genotype information of required number of mothers or fathers is generated randomly across 262,264 SNPs. After the mother or father genotype information at a SNP is generated, to be consistent with the trio part, the genotypes of an offspring for 98% or 99% of 262,264 SNPs are generated under *H*_0_. This time, higher probabilities are assigned to the offspring genotypes, whose family contributes to (1) as *N*_*M*=*C*_ = 1 (or *N*_*F*=*C*_ = 1), as seen in Table 3. For example, observing the genotype of mother or father as AA leads us to give 0.7 as the probability of obtaining an offspring with AA genotype and 0.3 as the probability of obtaining an offspring with AB genotype.

Lastly, the genotypes of an offspring at 1% or 2% of 262,264 SNPs are generated under *H*_1_, and offspring genotypes whose family contributes to (1) as *N*_*M*<*C*_ = 1 (or *N*_*F*<*C*_ = 1) are assigned with higher probabilities, as seen in Table 3. For example, observing the genotype of mother or father as AB, we assigned 0.7 as the probability of obtaining an offspring with AA genotype and 0.15 as the probability of obtaining an offspring with AB or BB genotype.

After obtaining the genotype information for the required number of trios and pairs across 262,264 SNPs for each simulation repetition of any given sample size, we treat this simulated genome-wide genotype data as if it is the original data and then apply permutation procedure defined in Section 2.2. 

However, simultaneously testing of 262,264 SNPs raises multiple testing issue in GWAS. One possible solution to this problem is Benjamini-Hochberg correction procedure [20]. For that reason, under each sample size, all 262,264 *P*  values are corrected by this procedure. Upon considering genome-wide significance level as 0.05, for each sample size, the corrected *P*  values which are resulting from SNPs generated under *H*_0_ are compared with this significance level, and false positive rate is computed with the formula given in

(5) False  Positive  Rate=Estimated  Type  I  Error  Rate=P^(Reject  H0 ∣ H0  is  true)=#(P value≤0.05)#SNPs  generated  under  H0,

where the numbers of SNPs generated under *H*_0_ are 257,019 and 259,640 for 98% and 99% scenarios, respectively.

Similarly, for each sample size, the corrected *P*  values from SNPs generated under *H*_1_ are compared with that significance level to compute false negative rate and, in turn, true positive rate with the formulas given in the following:

(6) False  Negative  Rate=Estimated  Type  II  Error  Rate=P^(Fail  to  reject  H0 ∣ H1  is  true)=#(P-value>0.05)#SNPs  generated  under  H1,

where the numbers of SNPs generated under *H*_1_ are 2,622 and 5,245 for 1% and 2% scenarios, respectively,

(7) True  Positive  Rate=Estimated  Power=P^(Reject  H0 ∣ H1  is  true)=1−False  Negative  Rate.

## 3. Results and Discussion

The power and the expected false positive rates are estimated under different sample sizes. All results are reported in Tables 4 and 5. 

Our simulation study shows that C-TDT is quite powerful. When only 1% of the SNPs are expected to be associated with the disease in the genome, as Table 4 reveals, C-TDT results in a 91% power even when there are 30 trios and 18 pairs. As expected, as the size of the sample increases, the power of the test increases and the estimated false positive rate decreases.

On the other hand, when only 2% of the SNPs are expected to be associated with the disease, it is possible to obtain an impressive amount of power, such as 99%, but this time if the budget and circumstances allow for more than hundred trios together with at least 65 pairs. It is especially promising to see that 50 trios and 30 pairs are enough in size to exceed an 80% amount of power. Furthermore, it is observed that while increasing the sample size from 50 trios and 30 pairs to 70 trios and 40 pairs results in an increased power to 92%, it leads to only a slight change in the estimated false positive rate. As a final note, although C-TDT could not attain a desirable amount of power for the two smallest sizes (10 trios and 4 pairs or 30 trios and 18 pairs) when the expected percentage of SNPs associated with the disease is 2%, it is clear that this test statistic is efficient even for small number of families when the expected number of SNPs associated with the disease in the genome is smaller. 

## 4. Conclusion

A common question in the planning stage of an experiment is either the attainable power for a predetermined sample size or the required sample size to attain a certain power. It is well known that collecting more data is beneficial in terms of statistical power. However, sometimes the cost of collecting additional information is greater than the benefit. In some cases, such as in late-onset complex diseases, it might be even impossible to attain these additional samples. Moreover, one of the parental genotype information might be missing in the limited sample that is collected. The statistical power of the tests is especially an important issue in such small sized studies. 

Not discarding case-parent pairs from the statistical analysis always results in statistical power gain compared to the studies restricted only to case-parent trios. For that reason, genotype information of case-parent pairs should be incorporated into the genetic association studies in an efficient way. One of the approaches would be the use of C-TDT test statistic. The simulation studies in Hu and Zhou [7] showed that the use of C-TDT increases the statistical power in the presence of both trios and pairs, compared to TDT using only trios. In addition to this, our simulation results show that C-TDT is an efficient test statistic such that it gives results with high power even in moderately small sized samples, which is an advantage over the test statistics requiring large numbers of families.

In this paper, the power and the false positive rate calculations are extended for thousands of SNPs. The simulation study is held in C programming language and the code is available for researchers to use in their own studies. 

One should note that C-TDT cannot be applied to the family-based association studies consisting of more than one affected offspring or consisting of only case-mother pairs or only case-father pairs [7]. Furthermore, it works under missing completely at random (MCAR) assumption such that it requires that the missingness status of a parent is independent of his/her genotype. Relaxation of these restrictions would require further work. 

## Figures and Tables

**Table 1:** Possible genotypes for an offspring.

	Mother genotype	Father genotype	Offspring genotype
1	AA	AA	AA
2	AA	AB	AA AB
3	AA	BB	AB
4	AB	AA	AA AB
5	AB	AB	AA AB AB BB
6	AB	BB	AB BB
7	BB	AA	AB
8	BB	AB	AB BB
9	BB	BB	BB

**Table 2:** Contribution of mother and father genotypes to *T* and *NT* terms in C-TDT statistic.

	Mother genotype	Father genotype	Offspring genotype	*T*	*NT*
1	AA	AA	AA	0	0
2	AA	AB	AA	1	0
3	AA	AB	AB	0	1
4	AA	BB	AB	0	0
5	AB	AA	AA	1	0
6	AB	AA	AB	0	1
7	AB	AB	AA	2	0
8	AB	AB	AB	1	1
9	AB	AB	BB	0	2
10	AB	BB	AB	1	0
11	AB	BB	BB	0	1
12	BB	AA	AB	0	0
13	BB	AB	AB	1	0
14	BB	AB	BB	0	1
15	BB	BB	BB	0	0

**Table 3:** Contribution of mother or father genotypes to *N*_*M*>*C* or *F*>*C*_, *N*_*M*<*C* or *F*<*C*_, and *N*_*M*=*C* or *F*=*C*_ terms in C-TDT statistic.

	Mother or father genotype	Offspring genotype	Frequency	*N*_*M*>*C* or *F*>*C*_	*N*_*M*<*C* or *F*<*C*_	*N*_*M*=*C* or *F*=*C*_
1	AA	AA	2	0	0	1
2	AA	AB	2	1	0	0
3	AB	AA	2	0	1	0
4	AB	AB	4	0	0	1
5	AB	BB	2	1	0	0
6	BB	AB	2	0	1	0
7	BB	BB	2	0	0	1

**Table 4:** Power and estimated probability of type I error for C-TDT across different sample sizes when 1% of the SNPs are generated under *H*_1_.

Family group type		Power (%)	Estimated probability of type I error (%)
Case-parent trio size	Case-parent pair size		
10	4	61.8	14.4
30	18	**91.3**	**8.9**
50	30	98.6	7.8
70	40	99.8	7.3
90	55	100.0	6.9
110	65	100.0	6.7
130	75	100.0	6.6

**Table 5:** Power and estimated probability of type I error for C-TDT across different sample sizes when 2% of the SNPs are generated under *H*_1_.

Family group type		Power (%)	Estimated probability of type I error (%)
Case-parent trio size	Case-parent pair size		
10	4	41.3	14.4
30	18	66.1	8.9
50	30	**83**	**7.8**
70	40	92	7.3
90	55	96.5	6.9
110	65	98.5	6.7
130	75	99.4	6.6
